# Patient-reported treatment burden, communication, and persistence intention during add-on peginterferon alfa-2b therapy for chronic hepatitis B: a cross-sectional study

**DOI:** 10.3389/fpubh.2026.1972004

**Published:** 2026-10-20

**Authors:** Ning Han, Huan Liu, Libo Yan, Hong Tang

**Affiliations:** 1 Center of Infectious Diseases, West China Hospital of Sichuan University, Chengdu, Sichuan, China; 2 Laboratory of Infectious and Liver Diseases, Institution of Infectious Diseases, West China Hospital of Sichuan University, Chengdu, Sichuan, China; 3 Division of Infectious Diseases, State Key Laboratory of Biotherapy, Sichuan University, Chengdu, Sichuan, China

**Keywords:** chronic hepatitis B, patient-reported outcome, peginterferon alfa-2b, physician-patient communication, psychological distress, treatment burden, treatment persistence

## Abstract

**Background:**

Add-on peginterferon alfa-2b (Peg-IFN) may help reduce hepatitis B surface antigen levels and achieve a functional cure in selected patients with chronic hepatitis B (CHB), but the treatment burden can make a finite course difficult to complete. We examined the patient-reported factors associated with thoughts of discontinuation and intentions to persist with treatment during add-on Peg-IFN therapy.

**Methods:**

This anonymous cross-sectional online survey used outcome-specific analytic populations. All 412 eligible respondents were included in discontinuation-related analyses, and the primary persistence analysis included 289 respondents who were still receiving Peg-IFN. The main outcomes included whether respondents had ever wanted to stop Peg-IFN due to adverse effects and a four-item persistence/value intention score. Psychological distress was assessed using the Patient Health Questionnaire-4 (PHQ-4).

**Results:**

Among 412 eligible respondents, 175 (42.5%) had wanted to stop Peg-IFN because of adverse effects, 136 (33.0%) had asked a physician about dose reduction or discontinuation, and 39 (9.5%) reported actual premature discontinuation. In the adjusted analysis, adverse-effect burden (odds ratio 1.86 per standard deviation (SD), 95% confidence interval 1.37–2.51) and the PHQ-4 score (odds ratio 1.76, 95% confidence interval 1.28–2.41) were associated with a history of adverse-effect-related discontinuation thoughts. Among 289 currently treated respondents, treatment belief (*β* = 0.280, 95% confidence interval 0.198–0.362) and physician–patient communication (*β* = 0.098, 95% confidence interval 0.017–0.179) were associated with higher persistence/value intention, while the PHQ-4 score was inversely associated with this outcome (*β* = −0.081, 95% confidence interval −0.148 to −0.013).

**Conclusion:**

Adverse-effect burden and current psychological distress were associated with a history of adverse-effect-related discontinuation thoughts, whereas treatment belief and physician–patient communication were associated with higher persistence/value intention among patients still receiving therapy. Prospective studies are needed to evaluate whether structured education, symptom and distress screening, and tiered follow-up improve the rates of treatment completion.

## Introduction

Chronic hepatitis B (CHB) remains a major global health problem despite effective antiviral therapy (1). Long-term nucleos(t)ide analog (NA) treatment can maintain virological suppression in the majority of adherent patients, but hepatitis B surface antigen (HBsAg) loss remains uncommon during indefinite suppressive therapy (2, 3). HBsAg decline and sustained HBsAg loss are important steps toward functional cure (4, 5). The benefit of functional-cure-oriented treatment depends not only on biological responses but also on whether patients can understand, tolerate, and complete the planned course. Add-on peginterferon alfa-2b (Peg-IFN) is used in selected patients receiving oral antiviral therapy to promote HBsAg decline and increase the possibility of functional cure (6, 7). Peg-IFN provides a finite immunomodulatory strategy, but fatigue, flu-like symptoms, injection-site reactions, dermatological symptoms, alopecia, appetite change, sleep disturbance, mood symptoms, and concerns about hematological abnormalities can make treatment difficult to sustain (8–10). Patients already stable on oral antiviral treatment must therefore weigh additional discomfort against a possible long-term benefit.

Treatment persistence is shaped by more than physical tolerability. Beliefs about the need for treatment, concerns about harm, and communication with clinicians have been associated with adherence across long-term conditions (11, 12). Interferon treatment can also affect quality of life in CHB (13). However, the majority of studies on add-on Peg-IFN have concentrated on virological, biochemical, or serological outcomes (6, 7, 14). Less is known about which patient-reported experiences accompany discontinuation thoughts and which potentially modifiable forms of support are related to a willingness to persist.

We conducted an anonymous cross-sectional survey of patients with CHB who were receiving or had completed add-on Peg-IFN during long-term NA therapy. The analysis examined two related patterns. Adverse-effect burden and psychological distress were evaluated in relation to discontinuation thoughts, while treatment beliefs and physician–patient communication were evaluated in relation to persistence/value intention. Psychological distress was considered in both models, and HBsAg-related feedback was treated as part of the treatment context (Figure 1A).

**Figure 1:**
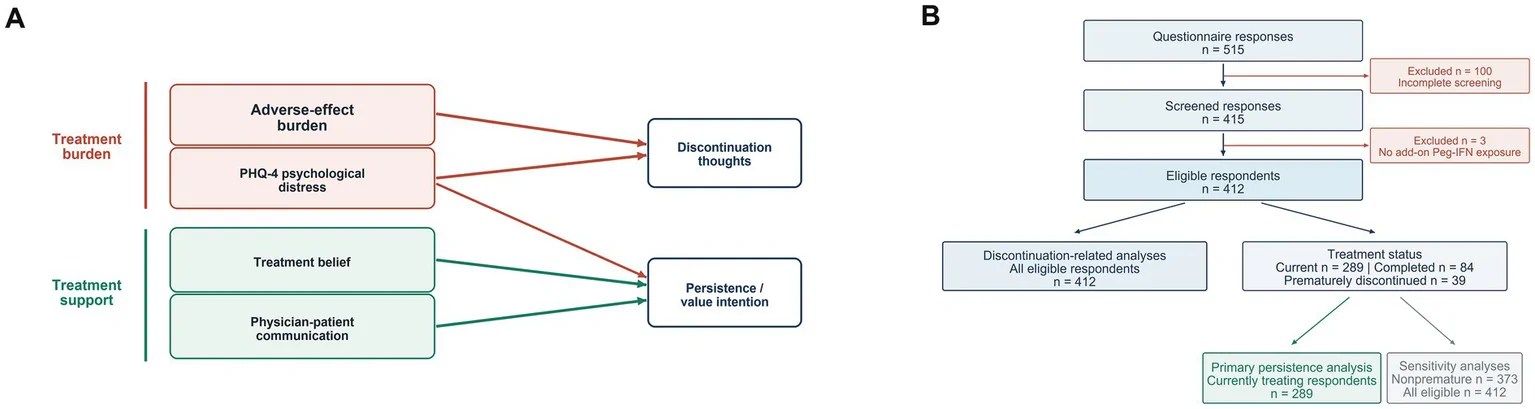
Analytical model and study flow. **(A)** Analytical model showing the patient-reported domains evaluated in relation to discontinuation thoughts and persistence/value intention. PHQ-4 psychological distress was included in both outcome models. Arrows represent modeled associations and do not imply causality. **(B)** Flow diagram showing the all-eligible population for discontinuation-related analyses and the currently treated population for the primary persistence-intention analysis. *n*, number of respondents; Peg-IFN, peginterferon alfa-2b; PHQ-4, Patient Health Questionnaire-4.

## Materials and methods

### Study design and participants

This anonymous cross-sectional online questionnaire study was conducted from 1 May to 13 May 2026, and is reported in accordance with the Strengthening the Reporting of Observational Studies in Epidemiology (STROBE) guidelines for cross-sectional studies (15). The questionnaire link was distributed through hospital-based WeChat patient follow-up groups by members of the clinical team. Since the number of patients who received or viewed the questionnaire link could not be determined, a formal response rate could not be calculated. Participation was voluntary. Respondents read the study information and provided electronic informed consent before beginning the questionnaire, and no direct personal identifiers were collected. The questionnaire received 515 submissions. Eligibility was assessed using items on physician-diagnosed CHB, current or previous long-term oral antiviral therapy, add-on Peg-IFN exposure, consent, and truthful self-completion. Among 412 eligible respondents, 289 were currently receiving Peg-IFN, 84 had completed the recommended course, and 39 had discontinued prematurely (Figure 1B). All 412 respondents were included in the primary discontinuation-related analyses. Because the persistence items asked about willingness and confidence to complete treatment, the 289 currently treated respondents formed the primary population for persistence analyses. The 373 respondents who had not discontinued prematurely and all 412 eligible respondents were used in sensitivity analyses of persistence intention. All eligible responses obtained during the survey period were considered for analysis.

### Questionnaire development and measures

The questionnaire was developed for this study to capture treatment experiences that are not routinely represented by virological or serological outcomes (16, 17). Items reflected issues encountered during Peg-IFN follow-up, including symptoms and treatment concerns, understanding of treatment goals, HBsAg-related feedback, and communication about benefit, risk, and adverse-effect management. The study team reviewed the questionnaire for clinical relevance, but it was not externally validated or formally pilot-tested before distribution. Except for the PHQ-4, the resulting domain scores were study-specific and were not treated as externally validated scales. Domain definitions, item mapping, scoring, and analytic roles are summarized in Supplementary Table S1, and the complete administered questionnaire is provided in Supplementary File S1. Adverse-effect burden was calculated as the mean of Q23–Q37, which were scored from 1 to 4. The resulting score was treated as a heterogeneous summary index rather than a validated unidimensional scale. Treatment belief was calculated as the mean of Q38–Q42, and physician–patient communication as the mean of Q43–Q45; both used five-point response scales. Higher values indicated greater burden, stronger treatment belief, or better communication, respectively. Persistence/value intention was calculated from Q54–Q57. These four items assessed willingness and confidence to complete treatment, perceived value in continuing, and willingness to tolerate discomfort for possible benefit. Psychological distress was assessed using the Patient Health Questionnaire-4 (PHQ-4), using its standard 2-week time frame (18, 19). Therefore, the PHQ-4 reflected psychological symptoms during the 2 weeks preceding the completion of the questionnaire. The four items assessed anxiety, uncontrolled worry, loss of interest, and depressed mood. Each item was scored from 0 to 3 and summed to a total score of 0–12. Scores of 0–2, 3–5, 6–8, and 9–12 were classified as normal, mild, moderate, and severe, respectively. The PHQ-4 was used as a brief symptom screening tool rather than a psychiatric diagnosis.

### HBsAg-related treatment feedback

HBsAg-related feedback was assessed from respondent reports of whether physicians had explained that add-on Peg-IFN was intended to promote HBsAg decline or clinical cure, whether the respondent knew the most recent HBsAg value or trend, and whether a physician had communicated that HBsAg had declined. These items were treated as recalled treatment and communication context rather than as laboratory-verified efficacy endpoints. For regression, physician-communicated HBsAg decline was coded as 0 for no decline or unclear feedback, 1 for decline of uncertain magnitude, and 2 for an obvious decline. A sensitivity analysis modeled these categories using indicator variables.

### Outcomes

The primary discontinuation-related outcome was whether a respondent had ever wanted to stop Peg-IFN because of adverse effects. Responses were dichotomized as never versus occasionally or often. Actual premature discontinuation, determined from reported treatment status, was examined as an additional clinically meaningful outcome. This was an all-cause outcome because respondents could select adverse effects, limited HBsAg response, physician recommendation, inconvenience, cost, or other reasons. The persistence-related outcome was the mean Q54–Q57 persistence/value intention score, ranging from 1 to 5. Higher scores indicated stronger willingness, confidence, and perceived value in completing treatment.

### Statistical analysis

Continuous variables were summarized with means and standard deviations (SDs) or medians and interquartile ranges, as appropriate. Categorical variables were summarized with counts and percentages. Internal consistency was assessed using Cronbach’s alpha. Exploratory measurement analyses of the adverse-effect items used polychoric correlations. Parallel analysis was used to determine the number of components, followed by minimum-residual exploratory factor analysis with promax rotation. Scores for the retained components were examined separately and jointly in sensitivity models for adverse-effect-related discontinuation thoughts. These analyses described the empirical item structure in this sample and were not treated as formal validation of the study-specific measures. Complete factor loadings and component-specific models are presented in Supplementary Table S2. Treatment beliefs and persistence/value intentions were compared using inter-domain Spearman correlations, the heterotrait-monotrait ratio, and one-factor and correlated two-factor ordinal models. Exploratory item loadings were also examined, with the corresponding measurement diagnostics reported in Supplementary Table S3. To reduce conceptual overlap, a sensitivity analysis replaced the five-item treatment-belief score with the more narrowly defined Q38–Q40 treatment-understanding score. For descriptive presentation of individual adverse-effect items, a score of 3 or 4 defined moderate-to-severe burden. The online questionnaire required completing the analytic items before submission, so the variables used in the primary models had no missing values.

Multivariable logistic regression for discontinuation thoughts included all 412 eligible respondents and standardized adverse-effect burden, standardized PHQ-4 total score, physician-communicated HBsAg decline, duration of oral antiviral therapy, duration of Peg-IFN therapy, age, and sex. The results are reported as odds ratios (ORs) with 95% confidence intervals (CIs). Actual premature discontinuation was examined in a parsimonious exploratory logistic regression model containing standardized adverse-effect burden, standardized PHQ-4 total scores, age, and sex. Treatment-duration variables were omitted from this model because premature discontinuation could itself shorten treatment duration.

Multivariable linear regression for persistence/value intention included the 289 respondents who were still receiving Peg-IFN. The model included standardized treatment belief, standardized physician–patient communication, standardized PHQ-4 total scores, standardized adverse-effect burden, physician-communicated HBsAg decline, duration of oral antiviral therapy, duration of Peg-IFN therapy, age, and sex. Heteroskedasticity-consistent type 3 (HC3) robust standard errors were used for the primary inference because the outcome was bounded and concentrated toward the upper end of the scale. Variance inflation factors were used to assess multicollinearity. The treatment-duration variables were entered as ordinal covariates in the primary models. Respondents who reported an unclear duration of oral antiviral treatment (*n* = 4) or Peg-IFN treatment (*n* = 8) were assigned the cohort median category. Sensitivity analyses of persistence/value intention were performed in the 373 respondents who had not discontinued prematurely and in all 412 eligible respondents. Additional sensitivity analyses adjusted for education, modeled treatment duration categorically, excluded respondents with unclear treatment duration, modeled HBsAg feedback using indicator variables, and used fractional-logit regression for the bounded persistence/value outcome. All statistical tests were two-sided, and a *p*-value of < 0.05 was considered statistically significant. Analyses were performed using R version 4.6.0.

## Results

### Participant characteristics

Among the 515 submitted questionnaires, 412 respondents met the eligibility criteria for add-on Peg-IFN exposure and were included in the discontinuation-related analyses (Figure 1B). Their median age was 40 years, and 256 (62.1%) were men. At the time of the survey, 289 respondents (70.1%) were receiving Peg-IFN, 84 (20.4%) had completed the recommended course, and 39 (9.5%) had discontinued prematurely. The currently treated subgroup had a median age of 40 years, and 183 (63.3%) were men. Participant characteristics are shown in Table 1.

**Table 1:** Participant characteristics by analytic population.

Characteristic	All eligible (*n* = 412)	Currently receiving Peg-IFN (*n* = 289)
Age, years	40 (36–48)	40 (35–47)
Male sex	256 (62.1)	183 (63.3)
Highest educational level		
Junior high school or below	74 (18.0)	40 (13.8)
Senior high school or technical secondary school	82 (19.9)	58 (20.1)
College or undergraduate degree	222 (53.9)	166 (57.4)
Graduate degree or above	34 (8.3)	25 (8.7)
Marital status		
Unmarried	47 (11.4)	36 (12.5)
Married	351 (85.2)	240 (83.0)
Divorced or widowed	14 (3.4)	13 (4.5)
Current work status		
Employed	283 (68.7)	209 (72.3)
Unemployed or not working	55 (13.3)	33 (11.4)
Retired	20 (4.9)	12 (4.2)
Student	2 (0.5)	2 (0.7)
Other	52 (12.6)	33 (11.4)
Current residence		
Same city	339 (82.3)	240 (83.0)
Same province, another city	64 (15.5)	43 (14.9)
Another province	9 (2.2)	6 (2.1)
One-way travel time to hospital		
<30 min	74 (18.0)	56 (19.4)
30–60 min	165 (40.0)	117 (40.5)
1–2 h	117 (28.4)	80 (27.7)
>2 h	56 (13.6)	36 (12.5)
Medical insurance		
Employee medical insurance	317 (76.9)	233 (80.6)
Resident medical insurance	56 (13.6)	34 (11.8)
Self-pay	35 (8.5)	20 (6.9)
Other	4 (1.0)	2 (0.7)
Duration of oral antiviral therapy		
<1 year	96 (23.3)	80 (27.7)
1–3 years	118 (28.6)	77 (26.6)
3–5 years	50 (12.1)	34 (11.8)
>5 years	144 (35.0)	95 (32.9)
Unclear	4 (1.0)	3 (1.0)
Current or recent oral antiviral drug		
Entecavir (ETV)	88 (21.4)	60 (20.8)
Tenofovir disoproxil fumarate (TDF)	48 (11.7)	34 (11.8)
Tenofovir alafenamide (TAF)	184 (44.7)	129 (44.6)
Combination therapy (ETV + TAF or ETV + TDF)	73 (17.7)	48 (16.6)
Unclear	19 (4.6)	18 (6.2)
Duration of add-on Peg-IFN therapy		
<12 weeks	103 (25.0)	76 (26.3)
12–24 weeks	115 (27.9)	82 (28.4)
25–48 weeks	121 (29.4)	86 (29.8)
>48 weeks	65 (15.8)	39 (13.5)
Unclear	8 (1.9)	6 (2.1)
Peg-IFN treatment status		
Currently receiving Peg-IFN	289 (70.1)	289 (100.0)
Completed as recommended	84 (20.4)	0 (0.0)
Prematurely discontinued	39 (9.5)	0 (0.0)

Values are *n* (%) unless otherwise stated. Age is presented as median (interquartile range). Percentages are calculated within each analytic population. ETV, entecavir; *n*, number of respondents; Peg-IFN, peginterferon alfa-2b; TAF, tenofovir alafenamide; TDF, tenofovir disoproxil fumarate.

### Questionnaire domain scores and internal consistency

Questionnaire scores and internal consistency estimates are shown in Table 2. In the all-eligible cohort, Cronbach’s alpha was 0.853 for the adverse-effect index and ranged from 0.801 to 0.923 for the other study-specific domains; The PHQ-4 alpha was 0.868. Values were similar in the currently treated subgroup. Moreover, 45 of 412 respondents (10.9%) and 30 of 289 currently treated respondents (10.4%) had moderate or severe PHQ-4 scores. Polychoric parallel analysis retained two components for the adverse-effect items. The first component was characterized mainly by sleep, mood, irritability, dermatological and injection-site effects, laboratory concerns, and interference with daily life. The second component was characterized mainly by fever or chills, fatigue, myalgia or arthralgia, and headache; appetite and weight items loaded on both. Full loadings are provided in Supplementary Table S2.

**Table 2:** Questionnaire scores and internal consistency by analytic population.

Domain	Items	Range	All eligible (*n* = 412), mean (SD); α	Currently receiving Peg-IFN (*n* = 289) Mean (SD); α
Adverse-effect burden	15	1–4	1.85 (0.45); 0.853	1.85 (0.43); 0.843
Treatment belief	5	1–5	3.93 (0.54); 0.801	4.00 (0.49); 0.787
Physician-patient communication	3	1–5	3.95 (0.55); 0.856	3.96 (0.54); 0.850
Persistence/value intention	4	1–5	4.03 (0.67); 0.923	4.15 (0.55); 0.923
PHQ-4 psychological distress	4	0–12	2.80 (2.39); 0.868	2.89 (2.32); 0.860

Values are presented as mean (SD). α denotes Cronbach’s alpha. *n*, number of respondents; Peg-IFN, peginterferon alfa-2b; PHQ-4, Patient Health Questionnaire-4; SD, standard deviation.

### Adverse-effect burden and discontinuation thoughts

Among all 412 eligible respondents, the most frequent moderate-to-severe adverse effects or concerns were fatigue (37.1%), rash or pruritus (28.4%), alopecia (27.4%), concern about leukocyte or platelet decline (24.8%), myalgia or arthralgia (24.0%), injection-site reactions (22.3%), reduced appetite (21.8%), worsened sleep (20.6%), irritability (18.9%), and weight loss (17.7%) (Figure 2; Supplementary Table S4). Overall, 175 respondents (42.5%) had wanted to stop Peg-IFN because of adverse effects, 136 (33.0%) had asked a physician about dose reduction or discontinuation, and 39 (9.5%) reported actual premature discontinuation.

**Figure 2:**
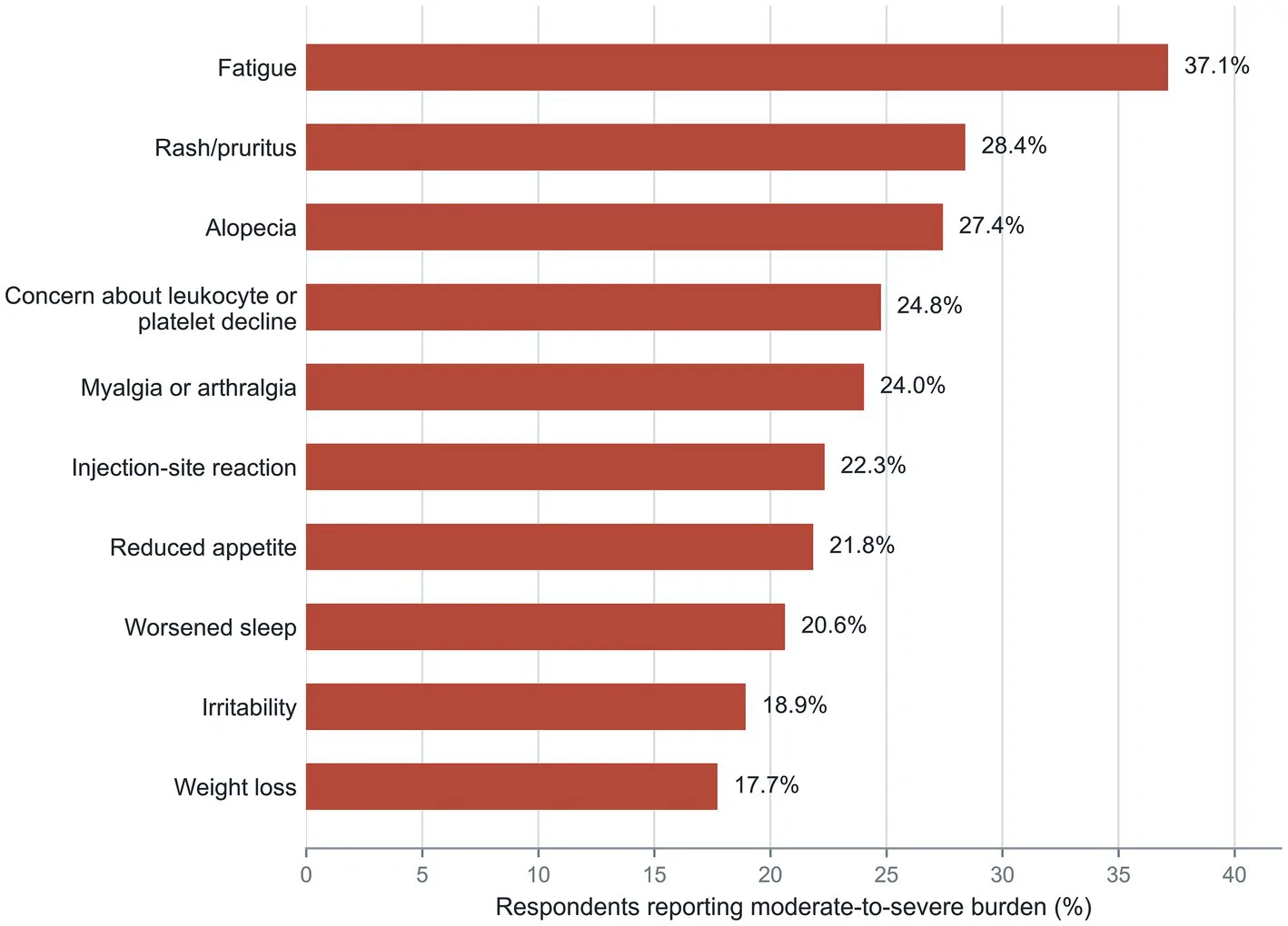
Most frequent moderate-to-severe patient-reported adverse effects. Bars show the percentage of all 412 eligible respondents who rated each adverse-effect item as moderate or severe.

### HBsAg-related treatment feedback

The majority of respondents recalled receiving an explanation of the treatment goal. Among all 412 eligible respondents, 400 (97.1%) reported being told that add-on Peg-IFN was intended to promote HBsAg decline or pursue clinical cure. Overall, 317 (76.9%) knew their most recent HBsAg value, and 395 (95.9%) knew either the value or its approximate trend. Physician-communicated HBsAg decline was reported by 322 respondents (78.2%), including 252 (61.2%) who reported an obvious decline and 70 (17.0%) who reported a decline of uncertain magnitude (Figure 3; Supplementary Table S5).

**Figure 3:**
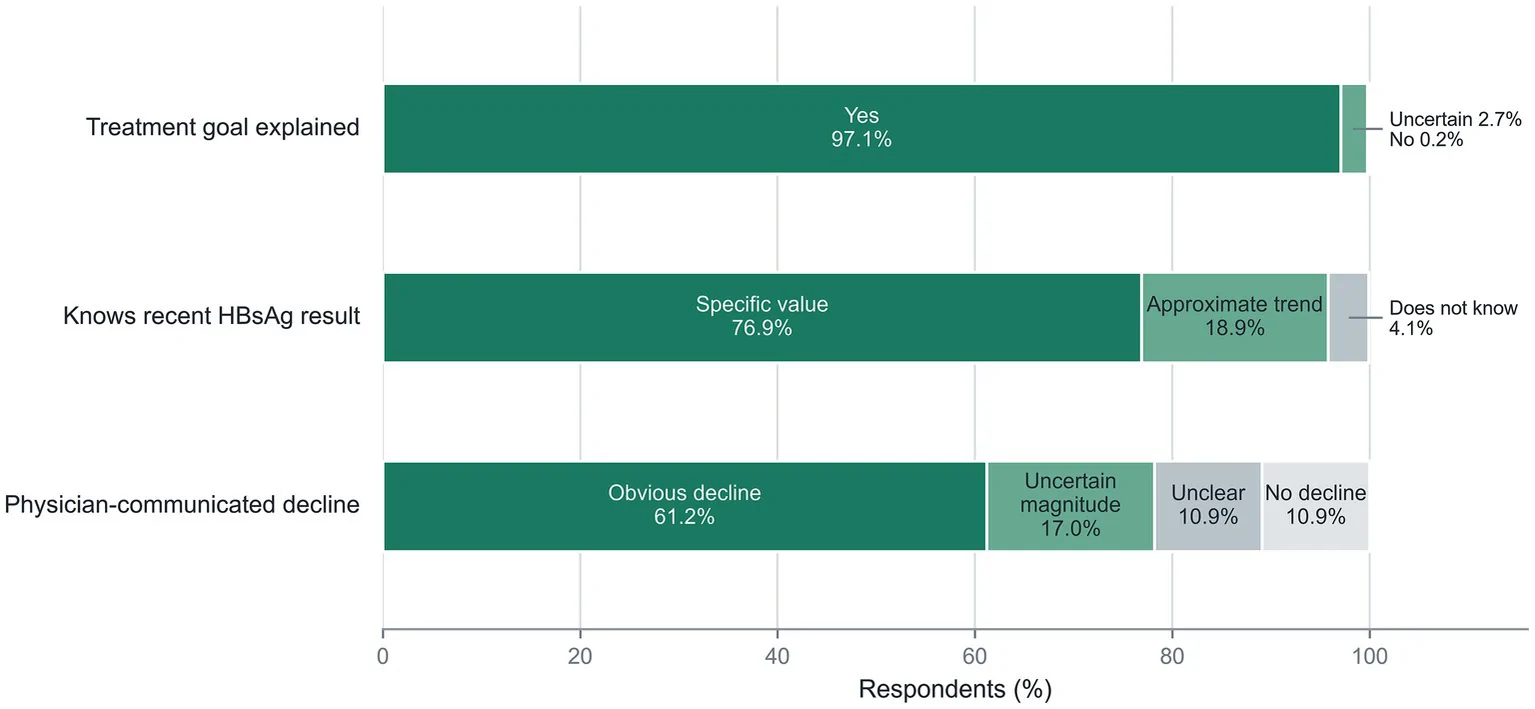
HBsAg-related treatment feedback among all 412 eligible respondents. The figure shows patient-reported knowledge of HBsAg results and recalled physician communication about HBsAg change. These data describe treatment context and are not laboratory-verified efficacy outcomes. HBsAg, hepatitis B surface antigen.

### Factors associated with discontinuation thoughts

The primary adjusted logistic regression model is shown in Table 3 and Figure 4A. Among all 412 eligible respondents, each SD increase in adverse-effect burden was associated with higher odds of adverse-effect-related discontinuation thoughts (OR 1.86, 95% CI 1.37–2.51, *p* < 0.001). A higher current PHQ-4 total score was also associated with a history of adverse-effect-related discontinuation thoughts (OR per SD 1.76, 95% CI 1.28–2.41, *p* < 0.001). HBsAg feedback, treatment duration, age, and sex were not independently associated with discontinuation thoughts. The results were similar in the 373 non-premature and 289 currently treated populations. In component-specific sensitivity analyses, both adverse-effect components were associated with discontinuation thoughts when entered separately (Component 1: OR 2.20, 95% CI 1.59–3.05; Component 2: OR 1.36, 95% CI 1.05–1.75). When both components were entered together, Component 1 remained associated (OR 2.16, 95% CI 1.51–3.09), whereas Component 2 did not remain associated (OR 1.04, 95% CI 0.78–1.38) (Supplementary Table S2). In the exploratory all-cause premature discontinuation model, total adverse-effect burden (OR 1.08, 95% CI 0.70–1.66) and the PHQ-4 score (OR 1.25, 95% CI 0.81–1.91) were not associated with reported premature discontinuation; older age was associated with higher odds (OR 1.05 per year, 95% CI 1.01–1.09). Additional discontinuation-related models and the reported reasons for premature discontinuation are presented in Supplementary Table S6.

**Table 3:** Multivariable logistic regression for discontinuation-related outcomes.

Outcome and predictor	*n* (events)	Adjusted OR (95% CI)	*p*-value
A. Adverse-effect-related discontinuation thoughts	412 (175)		
Adverse-effect burden (per SD)		1.86 (1.37–2.51)	< 0.001
PHQ-4 total score (per SD)		1.76 (1.28–2.41)	< 0.001
Physician-communicated HBsAg decline (ordinal)		1.19 (0.88–1.61)	0.261
Duration of oral antiviral therapy (ordinal)		0.88 (0.73–1.07)	0.214
Duration of add-on Peg-IFN therapy (ordinal)		1.03 (0.81–1.30)	0.825
Age (per year)		0.99 (0.96–1.02)	0.528
Male sex		0.77 (0.48–1.22)	0.259
B. Actual premature discontinuation (all-cause)	412 (39)		
Adverse-effect burden (per SD)		1.08 (0.70–1.66)	0.742
PHQ-4 total score (per SD)		1.25 (0.81–1.91)	0.315
Age (per year)		1.05 (1.01–1.09)	0.025
Male sex		0.82 (0.41–1.64)	0.574

Panel A modeled having ever wanted to stop Peg-IFN because of adverse effects. Panel B modeled reported all-cause premature discontinuation. Domain predictors and PHQ-4 were standardized. All listed predictors within each panel were entered simultaneously. CI, confidence interval; HBsAg, hepatitis B surface antigen; n, number of respondents; OR, odds ratio; Peg-IFN, peginterferon alfa-2b; PHQ-4, Patient Health Questionnaire-4; SD, standard deviation.

**Figure 4:**
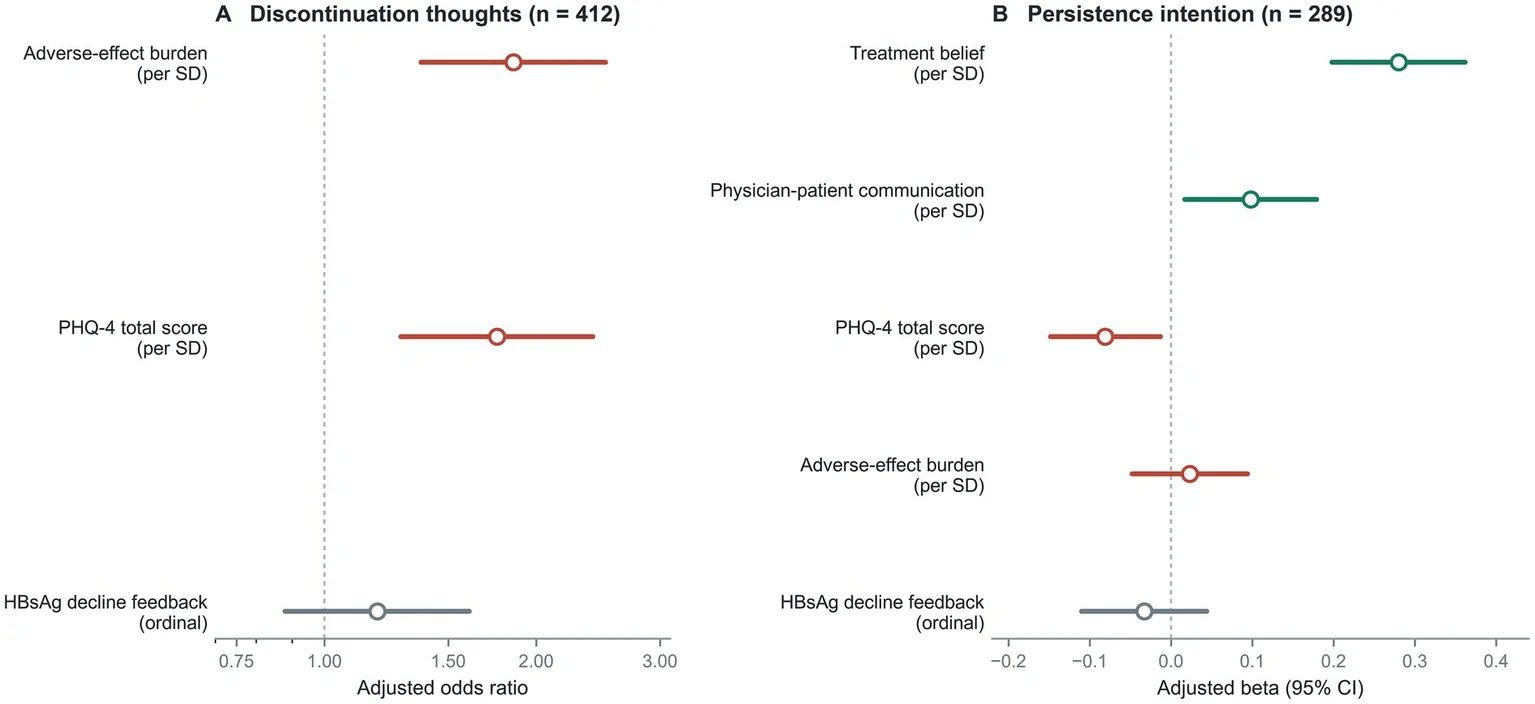
Key adjusted associations in the outcome-specific primary populations. **(A)** Adjusted ORs with 95% CIs from the logistic regression model for adverse-effect-related discontinuation thoughts among all 412 eligible respondents. **(B)** Adjusted regression coefficients with HC3 robust 95% CIs from the linear model for the Q54–Q57 persistence/value intention score among 289 currently treated respondents. Domain predictors and PHQ-4 scores were standardized. Full models and *p*-values are provided in Tables 3, 4. CI, confidence interval; HBsAg, hepatitis B surface antigen; HC3, heteroskedasticity-consistent type 3 robust standard errors; n, number of respondents; OR, odds ratio; PHQ-4, Patient Health Questionnaire-4; SD, standard deviation.

### Factors associated with persistence/value intention

The primary HC3 robust linear regression model among 289 currently treated respondents is shown in Table 4 and Figure 4B. Stronger treatment belief was associated with higher persistence/value intention (*β* = 0.280 per SD, 95% CI 0.198–0.362, *p* < 0.001). Better physician-patient communication was also associated with a higher score (*β* = 0.098, 95% CI 0.017–0.179, *p* = 0.018), while a higher PHQ-4 total score was associated with a lower score (*β* = −0.081, 95% CI − 0.148 to −0.013, *p* = 0.019). Adverse-effect burden and physician-communicated HBsAg decline were not independently associated with persistence/value intention. All variance inflation factors were 1.98 or lower (Supplementary Table S7).

**Table 4:** Multivariable linear regression for persistence/value intention among currently treated respondents.

Predictor	HC3 β (95% CI)	*p*-value
Treatment belief (per SD)	0.280 (0.198–0.362)	<0.001
Physician-patient communication (per SD)	0.098 (0.017–0.179)	0.018
PHQ-4 total score (per SD)	−0.081 (−0.148 to −0.013)	0.019
Adverse-effect burden (per SD)	0.023 (−0.048–0.094)	0.522
Physician-communicated HBsAg decline (ordinal)	−0.033 (−0.110–0.044)	0.405
Duration of oral antiviral therapy (ordinal)	−0.039 (−0.084–0.007)	0.094
Duration of add-on Peg-IFN therapy (ordinal)	0.025 (−0.033–0.083)	0.398
Age (per year)	0.002 (−0.004–0.007)	0.586
Male sex	0.020 (−0.091–0.131)	0.723

The outcome was the mean Q54–Q57 persistence/value intention score among 289 respondents who were still receiving Peg-IFN. Estimates and 95% CIs use HC3 robust standard errors. Domain predictors and PHQ-4 were standardized. CI, confidence interval; HBsAg, hepatitis B surface antigen; HC3, heteroskedasticity-consistent type 3 robust standard errors; Peg-IFN, peginterferon alfa-2b; PHQ-4, Patient Health Questionnaire-4; SD, standard deviation.

Persistence/value intention remained concentrated toward the upper end of the scale. Among the 289 currently treated respondents, 243 (84.1%) had a mean score of at least 4 and 61 (21.1%) had a score of 5 (Supplementary Table S7). Fractional-logit analysis retained the positive associations for treatment belief and physician–patient communication. The PHQ-4 estimate remained inverse, although its 95% CI included the null (Supplementary Table S8). Treatment belief and persistence/value intention were strongly related but not identical. In the currently treated subgroup, their Spearman correlation was 0.628, and the heterotrait-monotrait ratio was 0.782. A two-factor ordinal model fit better than a one-factor model, although the latent correlation was 0.798 and the root mean square error of approximation (RMSEA) remained elevated. Q41 and Q42 showed the greatest cross-loading in the exploratory analysis (Supplementary Table S3). When the treatment-belief score was restricted to the Q38–Q40 treatment-understanding items, the association with persistence/value intention remained (*β* = 0.203 per SD, 95% CI 0.134–0.272, *p* < 0.001) (Supplementary Table S9). The main associations were also broadly consistent in the 373 non-premature and 412 all-eligible sensitivity populations (Supplementary Table S9). The belief-by-communication interaction remained exploratory. It was nominally associated with persistence/value intention in the currently treated population but was not reproduced in the 373 non-premature or 412 all-eligible sensitivity populations. It was therefore not interpreted as stable effect modification (Supplementary Table S9).

## Discussion

This study identified two main patterns in patient-reported experience during add-on Peg-IFN therapy. Greater adverse-effect burden and current psychological distress were associated with a history of adverse-effect-related discontinuation thoughts. Among patients still receiving Peg-IFN, stronger treatment beliefs and better physician–patient communication were associated with higher persistence/value intention. Previous studies have mainly focused on virological response, safety, and treatment discontinuation (6, 7, 14, 20–22). Our findings add a patient-reported perspective by showing how treatment burden, distress, treatment belief, and communication relate to patients’ willingness to continue a demanding finite course.

Adverse-effect-related discontinuation thoughts were reported by 42.5% of respondents, whereas 9.5% reported actual premature discontinuation. Add-on Peg-IFN can improve functional-cure-related outcomes in selected patients, but treatment toxicity remains an important management issue (20, 21). In a prospective study of peginterferon alfa-2b, 22% of patients required dose reduction and 8% discontinued treatment (22). In our survey, both the adverse-effect burden index and the discontinuation-thought outcome referred to treatment adverse effects. The OR of 1.86 therefore quantifies the relationship between reported burden and thoughts of stopping because of adverse effects; it does not predict actual treatment discontinuation. In the exploratory all-cause model, adverse-effect burden was not associated with actual premature discontinuation, which reflected several reasons, including physician recommendation, perceived limited response, and tolerability. The actual discontinuation analysis was exploratory because only 39 events were available, and symptom burden was assessed at questionnaire completion rather than at the time treatment was stopped. Exploratory factor analysis supported two adverse-effect components rather than a single dimension. When both components were modeled together, only the broader component covering sleep and mood symptoms, dermatological and injection-site effects, laboratory concerns, and interference with daily life remained associated with discontinuation thoughts; these component labels require validation in other samples.

Psychological distress was related to both outcomes. Moderate or severe PHQ-4 scores were present in 10.9% of the all-eligible cohort. Previous research has linked treatment adherence with clinical outcomes (23), while studies on CHB have documented emotional and health-related quality-of-life burdens (24–26), including among patients receiving interferon (13). The PHQ-4 covered the preceding 2 weeks, whereas discontinuation thoughts could have occurred at any time during treatment. The observed association therefore links current distress to a history of discontinuation thoughts at the time of questionnaire completion. It does not show which occurred first or whether one contributed to the other. Repeated longitudinal assessments are needed to establish their temporal relationship.

Treatment belief had the largest adjusted association with persistence/value intention, with a 0.280-point increase in the outcome per SD increase in belief. Physician–patient communication showed a smaller association of 0.098 points. Treatment belief and persistence/value intention, however, shared substantial content. Their correlation and factor analyses supported partial rather than complete separation, with the greatest overlap observed for items concerning perceived treatment effectiveness and the chance of clinical cure. Part of the association may therefore reflect a shared positive appraisal of treatment. Nevertheless, the narrower Q38–Q40 treatment-understanding score remained associated with persistence/value intention, suggesting that understanding the purpose and meaning of treatment was also relevant to patients’ willingness to continue. This interpretation is broadly compatible with the necessity-concerns perspective, in which patients consider the perceived need for treatment together with concerns about harm (11). The communication finding is also consistent with a meta-analysis of 106 studies involving 45,093 patients, in which poor physician communication was associated with a 19% higher risk of non-adherence; communication training increased the odds of adherence across 21 intervention studies (12). Disease-specific studies have similarly linked treatment knowledge and related patient factors to adherence during oral hepatitis B virus (HBV) therapy (27, 28). HBsAg feedback was common in this study. Nearly 80% of respondents reported that their physician had told them that HBsAg had declined. After adjustment for other factors, reported HBsAg decline was not associated with discontinuation thoughts or persistence/value intention. HBsAg kinetics remain important markers of response to Peg-IFN therapy (29, 30). In this survey, the feedback was based on patient recall. Knowing that HBsAg had declined may not be enough to influence a patient’s willingness to continue treatment. A clear explanation of what the change means and how it affects the treatment plan may also be important. Laboratory results were not independently verified. Future prospective studies should include serial HBsAg results and assess how patients understand the information provided by their physicians.

These findings may inform the design of a scalable support pathway for Peg-IFN follow-up. Such a pathway could combine a brief assessment of adverse-effect burden and discontinuation thoughts, PHQ-4 screening, and structured explanation of treatment goals and adverse-effect management. Patients who report greater burden, psychological distress, or emerging discontinuation thoughts could receive more frequent follow-up or targeted support. This approach extends previous adherence work in routine CHB care (31) and is compatible with nurse-physician models that combine patient education with longitudinal disease management (32). Its effectiveness and cost-effectiveness should be evaluated prospectively.

This study has several strengths, including its focus on patient-reported experiences, use of the PHQ-4, and evaluation of the main associations across alternative cohorts and model specifications. Several limitations should also be considered. The cross-sectional design does not establish temporal order or causality. In particular, the PHQ-4 assessed symptoms during the preceding 2 weeks, whereas discontinuation thoughts could have occurred at any time during treatment. Current psychological distress may therefore not reflect the patient’s psychological state when discontinuation thoughts occurred. The recruitment through hospital-based WeChat follow-up groups may have preferentially included patients who were more engaged in follow-up, had more frequent communication with clinicians, or were more willing to continue treatment. Because the number of patients who received or viewed the questionnaire link was unknown, a formal response rate could not be calculated. The findings may therefore not represent all patients receiving add-on Peg-IFN, particularly those who are less engaged in hospital follow-up or online patient groups. The primary persistence analysis was restricted to respondents who were still receiving Peg-IFN, while respondents who had completed or discontinued treatment were included only in sensitivity analyses. Persistence/value intention nevertheless remained concentrated toward the upper end of the scale in the currently treated population, which may have limited discrimination between respondents with generally favorable intentions. Except for the PHQ-4, the study-specific measures were not externally validated or formally pilot-tested. The adverse-effect index contained two exploratory components and should therefore be interpreted as a heterogeneous summary of treatment burden rather than a validated unidimensional scale. Treatment belief and persistence/value intention also showed substantial shared variance, and part of their observed association may reflect measurement overlap.

Prospective studies should assess patient-reported burden, PHQ-4 symptoms, treatment belief, and communication at the start of Peg-IFN and at repeated follow-up visits. Linking these measures to verified HBsAg trajectories, missed injections, dose reduction, and premature discontinuation would establish temporal relationships and clinically meaningful thresholds. A subsequent intervention could compare usual care with standardized education, early symptom and distress screening, and tiered follow-up. Such work would determine whether the modifiable factors identified here can be translated into better treatment completion rather than remaining cross-sectional correlates.

## Conclusion

In this patient-reported survey, adverse-effect burden and current psychological distress were associated with a history of adverse-effect-related discontinuation thoughts. Among respondents still receiving treatment, stronger treatment belief and better physician–patient communication were associated with higher persistence/value intention. These findings support prospective evaluation of structured education, symptom and distress screening, and tiered follow-up during Peg-IFN treatment.

## Data Availability

The de-identified dataset generated and analyzed during the current study is available from the corresponding author on reasonable request, subject to institutional and ethical restrictions.
