# Supplementary material for: Patient-reported treatment burden, communication, and persistence intention during add-on peginterferon alfa-2b therapy for chronic hepatitis B: a cross-sectional study
Source: Front Public Health. 2026 Oct 20;14:1972004. doi: 10.3389/fpubh.2026.1972004 (PMC13727557; doi:10.3389/fpubh.2026.1972004)
Supplement: Supplementary file 1 [file Supplementary_file_1.docx]

# **Supplementary File 1. Complete online questionnaire**

| **Item** | **Original Chinese item** | **English translation** | **Response options or scoring** |
| --- | --- | --- | --- |
| Q1 | 我已阅读并理解上述内容，自愿参加本研究。 | I have read and understood the above information and voluntarily agree to participate in this study. | Yes; No |
| Q2 | 您是否由医生诊断为慢性乙型肝炎？ | Have you been diagnosed by a physician with chronic hepatitis B? | Yes; No |
| Q3 | 您是否正在或曾经长期口服抗病毒药物（恩替卡韦或替诺福韦）？ | Are you currently receiving or have you previously received long-term oral antiviral therapy with entecavir or tenofovir? | Yes; No |
| Q4 | 您是否正在口服抗病毒药基础上加用聚乙二醇干扰素（派格宾）治疗？ | Are you currently receiving peginterferon alfa-2b (PegBeron) in addition to oral antiviral therapy? | Yes, currently receiving treatment; Previously received and completed as recommended; Previously received but stopped prematurely; No |
| Q5 | 最近一次干扰素注射距今多久？ | How long has it been since your most recent interferon injection? | <=1 week; 1-4 weeks; 1-3 months; 3-6 months; >6 months |
| Q6 (screening confirmation) | 您确认以上信息真实，并愿意认真完成问卷吗？ | Do you confirm that the above information is true and that you are willing to complete the questionnaire carefully? | Yes |
| Q7 | 您的年龄:___岁 | Your age: ___ years | Free numeric entry |
| Q8 | 您的性别: | Sex: | Male; Female |
| Q9 | 您的最高学历: | Highest educational level: | Junior high school or below; Senior high school or technical secondary school; College or undergraduate degree; Graduate degree or above |
| Q10 | 您的婚姻状况: | Marital status: | Unmarried; Married; Divorced or widowed |
| Q11 | 您当前的工作状态: | Current employment status: | Employed; Unemployed; Retired; Student; Other |
| Q12 | 您目前居住地: | Current place of residence: | Same city; Same province but another city; Another province |
| Q13 | 您到医院单程大约需要多长时间? | Approximately how long does it take you to travel one way to the hospital? | <30 minutes; 30-60 minutes; 1-2 hours; >2 hours |
| Q14 | 您的医保类型: | Type of medical insurance: | Employee medical insurance; Resident medical insurance; Self-pay; Other |
| Q15 | 您口服核苷/核苷酸类似物抗病毒药物（恩替卡韦或替诺福韦）大约多久? | Approximately how long have you been taking oral nucleos(t)ide analogue antiviral therapy with entecavir or tenofovir? | <1 year; 1-3 years; 3-5 years; >5 years; Unclear |
| Q16 | 您当前或最近使用的口服抗病毒药物是: | Which oral antiviral drug are you currently using or most recently used? | Entecavir (ETV); Tenofovir disoproxil fumarate (TDF); Tenofovir alafenamide (TAF); Combination therapy (ETV+TAF or ETV+TDF); Unclear |
| Q17 | 您加用聚乙二醇干扰素治疗至今大约多久? | Approximately how long have you received add-on peginterferon therapy? | <12 weeks; 12-24 weeks; 25-48 weeks; >48 weeks; Unclear |
| Q18 | 医生是否告诉过您，加用聚乙二醇干扰素的主要目标是促进HBsAg下降或追求临床治愈? | Has your physician told you that the main goal of add-on peginterferon is to promote HBsAg decline or pursue clinical cure? | Yes; Uncertain; No |
| Q19 | 医生是否告诉过您预计治疗疗程? | Has your physician told you the expected treatment course? | Yes; Uncertain; No |
| Q20 | 医生是否告诉过您常见不良反应及处理方法? | Has your physician told you about common adverse effects and how to manage them? | Yes; Uncertain; No |
| Q21 | 您是否知道自己最近一次HBsAg，也就是乙肝表面抗原的结果或变化趋势? | Do you know your most recent HBsAg result or its trend? | Know the exact value; Know the approximate trend only; Do not know |
| Q22 | 医生是否告诉过您HBsAg较以前下降? | Has your physician told you that your HBsAg level has declined compared with before? | Marked decline; Declined but amount unclear; No decline; Unclear |
| Q23 | 发热或畏寒 | Fever or chills | None, Mild, Moderate, Severe; scored 1-4 |
| Q24 | 乏力 | Fatigue | None, Mild, Moderate, Severe; scored 1-4 |
| Q25 | 肌肉或关节酸痛 | Muscle or joint soreness/pain | None, Mild, Moderate, Severe; scored 1-4 |
| Q26 | 头痛 | Headache | None, Mild, Moderate, Severe; scored 1-4 |
| Q27 | 食欲下降 | Reduced appetite | None, Mild, Moderate, Severe; scored 1-4 |
| Q28 | 体重下降 | Weight loss | None, Mild, Moderate, Severe; scored 1-4 |
| Q29 | 脱发 | Alopecia | None, Mild, Moderate, Severe; scored 1-4 |
| Q30 | 睡眠变差 | Worsened sleep | None, Mild, Moderate, Severe; scored 1-4 |
| Q31 | 情绪低落 | Low mood | None, Mild, Moderate, Severe; scored 1-4 |
| Q32 | 易怒或烦躁 | Irritability or restlessness | None, Mild, Moderate, Severe; scored 1-4 |
| Q33 | 注射部位疼痛、红肿或硬结 | Injection-site pain, redness, swelling, or induration | None, Mild, Moderate, Severe; scored 1-4 |
| Q34 | 皮疹或瘙痒 | Rash or pruritus | None, Mild, Moderate, Severe; scored 1-4 |
| Q35 | 因白细胞或血小板下降而担心 | Concern about decreased leukocyte or platelet counts | None, Mild, Moderate, Severe; scored 1-4 |
| Q36 | 因甲状腺功能异常而担心 | Concern about thyroid dysfunction | None, Mild, Moderate, Severe; scored 1-4 |
| Q37 | 不良反应影响工作、学习或日常生活 | Adverse effects interfering with work, study, or daily life | None, Mild, Moderate, Severe; scored 1-4 |
| Q38 | 我清楚知道自己为什么要加用聚乙二醇干扰素。 | I clearly know why I am receiving add-on peginterferon. | Strongly disagree, Disagree, Neutral, Agree, Strongly agree; scored 1-5 |
| Q39 | 我理解HBsAg下降对治疗意味着什么。 | I understand what HBsAg decline means for treatment. | Strongly disagree, Disagree, Neutral, Agree, Strongly agree; scored 1-5 |
| Q40 | 我知道HBsAg下降和HBV DNA阴性不是同一件事。 | I know that HBsAg decline and HBV DNA negativity are not the same thing. | Strongly disagree, Disagree, Neutral, Agree, Strongly agree; scored 1-5 |
| Q41 | 我认为目前治疗对我有效。 | I believe my current treatment is effective. | Strongly disagree, Disagree, Neutral, Agree, Strongly agree; scored 1-5 |
| Q42 | 我认为自己有机会达到临床治愈。 | I believe I have a chance of achieving clinical cure. | Strongly disagree, Disagree, Neutral, Agree, Strongly agree; scored 1-5 |
| Q43 | 医生向我解释过HBsAg变化的意义。 | My physician has explained the meaning of HBsAg changes to me. | Strongly disagree, Disagree, Neutral, Agree, Strongly agree; scored 1-5 |
| Q44 | 医生向我解释过继续治疗的获益和风险。 | My physician has explained the benefits and risks of continuing treatment to me. | Strongly disagree, Disagree, Neutral, Agree, Strongly agree; scored 1-5 |
| Q45 | 医生告诉过我出现不良反应时该如何处理。 | My physician has told me how to manage adverse effects if they occur. | Strongly disagree, Disagree, Neutral, Agree, Strongly agree; scored 1-5 |
| Q46 | 即使有不良反应，如果HBsAg下降明显，我也愿意继续治疗。 | Even with adverse effects, I would be willing to continue treatment if HBsAg declined markedly. | Strongly disagree, Disagree, Neutral, Agree, Strongly agree; scored 1-5 |
| Q47 | 如果HBsAg没有明显下降，我更容易考虑停止聚乙二醇干扰素。 | If HBsAg has not declined markedly, I would be more likely to consider stopping peginterferon. | Strongly disagree, Disagree, Neutral, Agree, Strongly agree; scored 1-5 |
| Q48 | 过去4周是否漏打过聚乙二醇干扰素? | Have you missed any peginterferon injections in the past 4 weeks? | None; Once; >=2 times; Not applicable, treatment has been completed or stopped |
| Q49 | 过去4周是否延迟注射超过2天? | In the past 4 weeks, have you delayed an injection by more than 2 days? | None; Once; >=2 times; Not applicable, treatment has been completed or stopped |
| Q50 | 您是否按医生建议完成了Peg-IFN疗程？ | Have you completed the Peg-IFN course as recommended by your physician? | Currently still receiving treatment; Yes, completed; No, stopped prematurely |
| Q51 | 如果您曾提前停止Peg-IFN，主要原因是？ | If you stopped Peg-IFN prematurely, what was the main reason? | Adverse effects were difficult to tolerate; HBsAg decline was not obvious or treatment seemed ineffective; Physician advised stopping; Time or follow-up inconvenience; Cost; Other; multiple selections allowed |
| Q52 | 您是否曾因不良反应想停止聚乙二醇干扰素? | Have you ever wanted to stop peginterferon because of adverse effects? | Never; Occasionally; Often |
| Q53 | 您是否曾主动询问医生能否减量或停药? | Have you ever actively asked your physician whether the dose could be reduced or treatment stopped? | Yes; No |
| Q54 | 我愿意继续完成医生建议的聚乙二醇干扰素疗程。 | I am willing to continue and complete the peginterferon course recommended by my physician. | Strongly disagree, Disagree, Neutral, Agree, Strongly agree; scored 1-5 |
| Q55 | 我有信心完成医生建议的聚乙二醇干扰素疗程。 | I am confident that I can complete the peginterferon course recommended by my physician. | Strongly disagree, Disagree, Neutral, Agree, Strongly agree; scored 1-5 |
| Q56 | 我认为目前治疗值得继续。 | I think the current treatment is worth continuing. | Strongly disagree, Disagree, Neutral, Agree, Strongly agree; scored 1-5 |
| Q57 | 即使治疗过程不舒服，我仍愿意为了更好的疗效坚持。 | Even if treatment is uncomfortable, I am willing to persist for better efficacy. | Strongly disagree, Disagree, Neutral, Agree, Strongly agree; scored 1-5 |
|  | 在过去两周内，您有多少时间受到以下问题困扰？ | Over the last two weeks, how often have you been bothered by the following problems? |  |
| Q58 | 感到紧张、焦虑或急切。 | Feeling nervous, anxious, or on edge. | Not at all, Several days, More than half the days, Nearly every day; scored 0-3 |
| Q59 | 无法停止或控制担忧。 | Not being able to stop or control worrying. | Not at all, Several days, More than half the days, Nearly every day; scored 0-3 |
| Q60 | 做事时提不起劲或没有兴趣。 | Little interest or pleasure in doing things. | Not at all, Several days, More than half the days, Nearly every day; scored 0-3 |
| Q61 | 感到心情低落、沮丧或绝望。 | Feeling down, depressed, or hopeless. | Not at all, Several days, More than half the days, Nearly every day; scored 0-3 |
| Q62 | 您是否确认本问卷由您本人根据真实情况填写? | Do you confirm that this questionnaire was completed by yourself according to your true situation? | Yes; No |
