# Supplementary material for: Patient-reported treatment burden, communication, and persistence intention during add-on peginterferon alfa-2b therapy for chronic hepatitis B: a cross-sectional study
Source: Front Public Health. 2026 Oct 20;14:1972004. doi: 10.3389/fpubh.2026.1972004 (PMC13727557; doi:10.3389/fpubh.2026.1972004)
Supplement: Supplementary file 2 [file Supplementary_file_2.docx]

**Supplementary Tables**

**Supplementary Table 1. Questionnaire domains, scoring, and analytic role**

| **Domain or variable** | **Items** | **Response scale** | **Score derivation** | **Analysis role** |
| --- | --- | --- | --- | --- |
| Adverse-effect burden | Q23-Q37 | None to severe; 1-4 | Mean of 15 items | Primary exposure for discontinuation thoughts; heterogeneous burden index |
| Treatment belief | Q38-Q42 | Strongly disagree to strongly agree; 1-5 | Mean of 5 items | Primary exposure for persistence/value intention |
| Treatment understanding | Q38-Q40 | Strongly disagree to strongly agree; 1-5 | Mean of 3 items | Sensitivity exposure addressing overlap with persistence |
| Physician-patient communication | Q43-Q45 | Strongly disagree to strongly agree; 1-5 | Mean of 3 items | Primary exposure for persistence/value intention |
| Persistence/value intention | Q54-Q57 | Strongly disagree to strongly agree; 1-5 | Mean of 4 items | Primary continuous outcome among currently treated respondents |
| PHQ-4 psychological distress | Q58-Q61 | Past two weeks; 0-3 per item | Sum of 4 items; total 0-12 | Exposure in both primary models |
| HBsAg-related feedback | Q18-Q22 | Categorical | Q22 coded 0-2 | Treatment-context covariate |
| Treatment status | Q50-Q51 | Categorical and multiple choice | Current, completed, or premature discontinuation | Cohort definition and secondary actual-discontinuation outcome |
| Discontinuation thoughts | Q52 | Never, occasionally, often | Never vs occasionally/often | Primary binary discontinuation-related outcome |

**Supplementary Table 2. Exploratory structure of the adverse-effect burden items**

**Panel A. Two-component minimum-residual factor solution using polychoric correlations and promax rotation**

| **Item** | **Adverse-effect item** | **Component 1** | **Component 2** | **Uniqueness** |
| --- | --- | --- | --- | --- |
| Q23 | Fever/chills | -0.258 | 0.850 | 0.470 |
| Q24 | Fatigue | 0.146 | 0.701 | 0.367 |
| Q25 | Myalgia/arthralgia | -0.134 | 0.839 | 0.411 |
| Q26 | Headache | 0.070 | 0.654 | 0.514 |
| Q27 | Reduced appetite | 0.302 | 0.426 | 0.575 |
| Q28 | Weight loss | 0.264 | 0.277 | 0.767 |
| Q29 | Alopecia | 0.520 | -0.056 | 0.760 |
| Q30 | Worsened sleep | 0.643 | -0.002 | 0.588 |
| Q31 | Low mood | 0.770 | 0.107 | 0.299 |
| Q32 | Irritability | 0.726 | 0.046 | 0.431 |
| Q33 | Injection-site reaction | 0.507 | -0.067 | 0.778 |
| Q34 | Rash/pruritus | 0.625 | -0.191 | 0.713 |
| Q35 | Concern about leukocyte/platelet decline | 0.586 | 0.041 | 0.627 |
| Q36 | Concern about thyroid dysfunction | 0.536 | 0.081 | 0.655 |
| Q37 | Interference with work/study/daily life | 0.609 | 0.285 | 0.343 |

**Note.** Polychoric parallel analysis retained two components. Component 1 mainly reflected sleep, mood, irritability, dermatologic and injection-site effects, laboratory concerns, and daily-life interference. Component 2 mainly reflected acute systemic symptoms. The oblique component correlation was 0.590. Labels are descriptive and do not constitute external validation.

**Panel B. Component-score sensitivity analyses for adverse-effect-related discontinuation thoughts**

| **Model** | **Component** | **n (events)** | **Adjusted OR (95% CI)** | **P value** |
| --- | --- | --- | --- | --- |
| Adverse component 1 entered separately | Component 1 | 412 (175) | 2.20 (1.59-3.05) | <0.001 |
| Adverse component 2 entered separately | Component 2 | 412 (175) | 1.36 (1.05-1.75) | 0.019 |
| Both adverse components entered | Component 1 | 412 (175) | 2.16 (1.51-3.09) | <0.001 |
| Both adverse components entered | Component 2 | 412 (175) | 1.04 (0.78-1.38) | 0.806 |

**Supplementary Table 3. Treatment-belief and persistence/value-intention discriminant diagnostics**

**Panel A. Spearman correlations among questionnaire domains in all eligible respondents**

| **Domain** | **Adverse burden** | **PHQ-4** | **Treatment belief** | **Communication** | **Persistence intention** | **HBsAg feedback** |
| --- | --- | --- | --- | --- | --- | --- |
| Adverse burden | 1.000 | 0.664 | -0.165 | -0.135 | -0.164 | 0.070 |
| PHQ-4 | 0.664 | 1.000 | -0.148 | -0.153 | -0.203 | 0.018 |
| Treatment belief | -0.165 | -0.148 | 1.000 | 0.647 | 0.640 | 0.231 |
| Communication | -0.135 | -0.153 | 0.647 | 1.000 | 0.537 | 0.179 |
| Persistence intention | -0.164 | -0.203 | 0.640 | 0.537 | 1.000 | 0.132 |
| HBsAg feedback | 0.070 | 0.018 | 0.231 | 0.179 | 0.132 | 1.000 |

**Panel B. Correlation and HTMT summary**

| **Cohort** | **n** | **Spearman correlation** | **HTMT** | **Belief alpha** | **Persistence alpha** |
| --- | --- | --- | --- | --- | --- |
| All eligible | 412 | 0.640 | 0.785 | 0.801 | 0.923 |
| Currently receiving Peg-IFN | 289 | 0.628 | 0.782 | 0.787 | 0.923 |

**Panel C. Ordinal one-factor and two-correlated-factor model fit**

| **Cohort** | **Model** | **Scaled chi-square** | **df** | **CFI** | **TLI** | **RMSEA** | **SRMR** | **Latent correlation** |
| --- | --- | --- | --- | --- | --- | --- | --- | --- |
| All eligible | One factor | 757.2 | 27 | 0.960 | 0.946 | 0.257 | 0.092 |  |
| All eligible | Two correlated factors | 419.9 | 26 | 0.978 | 0.970 | 0.192 | 0.068 | 0.795 |
| Currently receiving Peg-IFN | One factor | 463.3 | 27 | 0.968 | 0.957 | 0.237 | 0.081 |  |
| Currently receiving Peg-IFN | Two correlated factors | 191.0 | 26 | 0.988 | 0.983 | 0.148 | 0.053 | 0.798 |

**Note.** The two-factor model fit better than the one-factor model, but the latent correlation remained high and RMSEA indicated residual misfit. These results support related but only partly distinct constructs.

**Panel D. Exploratory two-factor loadings for treatment-belief and persistence items**

| **Item** | **Intended domain** | **Factor 1** | **Factor 2** | **Uniqueness** |
| --- | --- | --- | --- | --- |
| Belief_Q38 | Treatment belief | 0.143 | 0.691 | 0.362 |
| Belief_Q39 | Treatment belief | -0.002 | 0.929 | 0.140 |
| Belief_Q40 | Treatment belief | -0.122 | 0.876 | 0.369 |
| Belief_Q41 | Treatment belief | 0.380 | 0.359 | 0.535 |
| Belief_Q42 | Treatment belief | 0.662 | 0.139 | 0.413 |
| Persistence_Q54 | Persistence/value intention | 0.976 | -0.055 | 0.120 |
| Persistence_Q55 | Persistence/value intention | 1.010 | -0.091 | 0.101 |
| Persistence_Q56 | Persistence/value intention | 0.965 | -0.052 | 0.136 |
| Persistence_Q57 | Persistence/value intention | 0.801 | 0.132 | 0.192 |

**Note.** Persistence items loaded mainly on Factor 1 and the first three treatment-belief items mainly on Factor 2. Q41 and Q42 showed greater cross-loading. Polychoric parallel analysis of all nine items retained one component, underscoring substantial shared variance.

**Supplementary Table 4. Adverse-effect burden and discontinuation-related outcomes among all eligible respondents**

| **Item or outcome** | **Mean (SD)** | **Moderate-to-severe or yes, n (%)** |
| --- | --- | --- |
| Fatigue | 2.23 (0.81) | 153 (37.1) |
| Rash/pruritus | 2.01 (0.91) | 117 (28.4) |
| Alopecia | 1.99 (0.95) | 113 (27.4) |
| Concern about leukocyte/platelet decline | 1.99 (0.80) | 102 (24.8) |
| Myalgia/arthralgia | 1.96 (0.79) | 99 (24.0) |
| Injection-site reaction | 1.92 (0.75) | 92 (22.3) |
| Reduced appetite | 1.86 (0.83) | 90 (21.8) |
| Worsened sleep | 1.89 (0.80) | 85 (20.6) |
| Irritability | 1.83 (0.76) | 78 (18.9) |
| Weight loss | 1.78 (0.78) | 73 (17.7) |
| Low mood | 1.78 (0.77) | 72 (17.5) |
| Interference with work/study/daily life | 1.72 (0.72) | 55 (13.3) |
| Headache | 1.64 (0.74) | 53 (12.9) |
| Fever/chills | 1.62 (0.74) | 49 (11.9) |
| Concern about thyroid dysfunction | 1.47 (0.68) | 39 (9.5) |
| Wanted to stop Peg-IFN because of adverse effects |  | 175 (42.5) |
| Asked physician about dose reduction or discontinuation |  | 136 (33.0) |
| Actual premature discontinuation |  | 39 (9.5) |

**Note.** Moderate-to-severe prevalence was defined as an item score of 3 or 4.

**Supplementary Table 5. HBsAg-related treatment feedback among all eligible respondents**

| **Item** | **Category** | **n** | **%** |
| --- | --- | --- | --- |
| Treatment goal explained as HBsAg decline or clinical cure | Yes | 400 | 97.1 |
|  | Uncertain | 11 | 2.7 |
|  | No | 1 | 0.2 |
| Knows recent HBsAg result or trend | Knows specific value | 317 | 76.9 |
|  | Knows approximate trend only | 78 | 18.9 |
|  | Does not know | 17 | 4.1 |
| Physician-communicated HBsAg decline | Obvious decline | 252 | 61.2 |
|  | Decline of uncertain magnitude | 70 | 17.0 |
|  | Unclear | 45 | 10.9 |
|  | No decline | 45 | 10.9 |

**Supplementary Table 6. Discontinuation-related models across analytic populations**

| **Model/cohort** | **n (events)** | **Predictor** | **Adjusted OR (95% CI)** | **P value** |
| --- | --- | --- | --- | --- |
| Discontinuation thoughts: all eligible | 412 (175) | Adverse-effect burden (per SD) | 1.86 (1.37-2.51) | <0.001 |
| Discontinuation thoughts: all eligible | 412 (175) | PHQ-4 total score (per SD) | 1.76 (1.28-2.41) | <0.001 |
| Discontinuation thoughts: all eligible | 412 (175) | HBsAg decline feedback (ordinal) | 1.19 (0.88-1.61) | 0.261 |
| Discontinuation thoughts: nonpremature sensitivity | 373 (147) | Adverse-effect burden (per SD) | 1.94 (1.39-2.70) | <0.001 |
| Discontinuation thoughts: nonpremature sensitivity | 373 (147) | PHQ-4 total score (per SD) | 1.66 (1.19-2.31) | 0.003 |
| Discontinuation thoughts: nonpremature sensitivity | 373 (147) | HBsAg decline feedback (ordinal) | 1.31 (0.94-1.81) | 0.106 |
| Discontinuation thoughts: Currently receiving Peg-IFN sensitivity | 289 (118) | Adverse-effect burden (per SD) | 1.90 (1.31-2.74) | <0.001 |
| Discontinuation thoughts: Currently receiving Peg-IFN sensitivity | 289 (118) | PHQ-4 total score (per SD) | 1.60 (1.11-2.32) | 0.013 |
| Discontinuation thoughts: Currently receiving Peg-IFN sensitivity | 289 (118) | HBsAg decline feedback (ordinal) | 1.49 (1.01-2.19) | 0.044 |
| Asked about dose reduction/stopping: all eligible | 412 (136) | Adverse-effect burden (per SD) | 1.24 (0.94-1.64) | 0.136 |
| Asked about dose reduction/stopping: all eligible | 412 (136) | PHQ-4 total score (per SD) | 1.28 (0.97-1.70) | 0.083 |
| Asked about dose reduction/stopping: all eligible | 412 (136) | HBsAg decline feedback (ordinal) | 1.31 (0.98-1.76) | 0.071 |

**Panel B. Reported reasons among 39 respondents with premature discontinuation**

| **Reason (multiple selections allowed)** | **n** | **% of 39** |
| --- | --- | --- |
| Physician recommended stopping | 22 | 56.4 |
| Adverse effects were difficult to tolerate | 17 | 43.6 |
| Limited HBsAg decline or perceived poor efficacy | 15 | 38.5 |
| Inconvenient timing or follow-up | 1 | 2.6 |
| Cost | 1 | 2.6 |
| Other | 4 | 10.3 |

**Supplementary Table 7. Persistence-model diagnostics and outcome concentration**

**Panel A. Variance inflation factors in the currently treated primary model**

| **Predictor** | **VIF** |
| --- | --- |
| Treatment belief | 1.98 |
| Physician-patient communication | 1.92 |
| PHQ-4 | 1.89 |
| Adverse-effect burden | 1.84 |
| HBsAg decline feedback | 1.35 |
| Oral antiviral duration | 1.07 |
| Peg-IFN duration | 1.38 |
| Age | 1.15 |
| Male sex | 1.09 |

**Panel B. Distribution and ceiling concentration of Q54-Q57 persistence/value intention**

| **Cohort** | **n** | **Mean (SD)** | **Median (IQR)** | **Score >=4, n (%)** | **Score =5, n (%)** |
| --- | --- | --- | --- | --- | --- |
| Currently receiving Peg-IFN | 289 | 4.15 (0.55) | 4.00 (4.00-4.25) | 243 (84.1) | 61 (21.1) |
| Nonpremature | 373 | 4.11 (0.58) | 4.00 (4.00-4.25) | 309 (82.8) | 71 (19.0) |
| All eligible | 412 | 4.03 (0.67) | 4.00 (4.00-4.25) | 321 (77.9) | 73 (17.7) |

**Supplementary Table 8. Fractional-logit sensitivity analysis for persistence/value intention among currently treated respondents**

| **Predictor** | **Coefficient (95% CI)** | **P value** | **Average marginal effect, score points** |
| --- | --- | --- | --- |
| Treatment belief | 0.451 (0.330 to 0.572) | <0.001 | 0.286 |
| Physician-patient communication | 0.194 (0.077 to 0.311) | 0.001 | 0.123 |
| PHQ-4 | -0.105 (-0.211 to 0.001) | 0.052 | -0.067 |
| Adverse-effect burden | 0.028 (-0.089 to 0.146) | 0.638 | 0.018 |
| HBsAg decline feedback | -0.084 (-0.212 to 0.044) | 0.197 | -0.053 |
| Oral antiviral duration | -0.059 (-0.131 to 0.013) | 0.110 | -0.037 |
| Peg-IFN duration | 0.046 (-0.050 to 0.142) | 0.346 | 0.029 |
| Age | 0.002 (-0.008 to 0.011) | 0.686 | 0.001 |
| Male sex | 0.049 (-0.124 to 0.222) | 0.577 | 0.031 |

**Note.** The Q54-Q57 score was rescaled from 1-5 to 0-1. HC3-type robust standard errors were used. Average marginal effects are shown on the original 1-5 score scale.

**Supplementary Table 9. Persistence/value-intention sensitivity analyses**

**Panel A. Key estimates across analytic populations**

| **Model/cohort** | **n** | **Predictor** | **HC3 beta (95% CI)** | **P value** |
| --- | --- | --- | --- | --- |
| Persistence intention: Currently receiving Peg-IFN primary | 289 | Treatment belief (per SD) | 0.280 (0.198 to 0.362) | <0.001 |
| Persistence intention: Currently receiving Peg-IFN primary | 289 | Physician-patient communication (per SD) | 0.098 (0.017 to 0.179) | 0.018 |
| Persistence intention: Currently receiving Peg-IFN primary | 289 | PHQ-4 total score (per SD) | -0.081 (-0.148 to -0.013) | 0.019 |
| Persistence intention: nonpremature sensitivity | 373 | Treatment belief (per SD) | 0.268 (0.148 to 0.388) | <0.001 |
| Persistence intention: nonpremature sensitivity | 373 | Physician-patient communication (per SD) | 0.137 (0.033 to 0.241) | 0.010 |
| Persistence intention: nonpremature sensitivity | 373 | PHQ-4 total score (per SD) | -0.088 (-0.155 to -0.020) | 0.011 |
| Persistence intention: all eligible sensitivity | 412 | Treatment belief (per SD) | 0.320 (0.196 to 0.443) | <0.001 |
| Persistence intention: all eligible sensitivity | 412 | Physician-patient communication (per SD) | 0.137 (0.027 to 0.247) | 0.014 |
| Persistence intention: all eligible sensitivity | 412 | PHQ-4 total score (per SD) | -0.084 (-0.153 to -0.015) | 0.017 |

**Panel B. Sensitivity analyses reducing conceptual overlap in treatment belief**

| **Model** | **Predictor** | **HC3 beta (95% CI)** | **P value** |
| --- | --- | --- | --- |
| Persistence intention: Q38-Q40 treatment-understanding sensitivity | Treatment understanding Q38-Q40 | 0.203 (0.134 to 0.272) | <0.001 |
| Persistence intention: Q38-Q40 treatment-understanding sensitivity | Physician-patient communication | 0.157 (0.088 to 0.225) | <0.001 |
| Persistence intention: Q38-Q40 treatment-understanding sensitivity | PHQ-4 | -0.083 (-0.151 to -0.015) | 0.017 |
| Persistence intention: treatment belief excluding Q42 | Treatment belief Q38-Q41 | 0.230 (0.153 to 0.308) | <0.001 |
| Persistence intention: treatment belief excluding Q42 | Physician-patient communication | 0.134 (0.058 to 0.209) | <0.001 |
| Persistence intention: treatment belief excluding Q42 | PHQ-4 | -0.083 (-0.151 to -0.015) | 0.017 |

**Panel C. Exploratory treatment-belief by communication interaction**

| **Cohort** | **n** | **HC3 beta (95% CI)** | **P value** |
| --- | --- | --- | --- |
| Currently receiving Peg-IFN | 289 | 0.040 (0.006 to 0.074) | 0.023 |
| Nonpremature | 373 | 0.018 (-0.044 to 0.080) | 0.572 |
| All eligible | 412 | -0.008 (-0.053 to 0.037) | 0.731 |

**Note.** The interaction was not stable across analytic populations and was not interpreted as robust effect modification.
